# Cosmetic outcome of femtosecond laser-assisted pterygium surgery

**DOI:** 10.1186/s40662-021-00230-w

**Published:** 2021-03-06

**Authors:** Darren Shu Jeng Ting, Yu-Chi Liu, Yi Fang Lee, Angel Jung Se Ji, Tien-En Tan, Hla M. Htoon, Jodhbir S. Mehta

**Affiliations:** 1 Academic Ophthalmology, Division of Clinical Neuroscience, School of Medicine, University of Nottingham, Nottingham, UK; 2 Department of Ophthalmology, Queen’s Medical Centre, Nottingham, UK; 3 Singapore Eye Research Institute, Singapore, Singapore; 4 Singapore National Eye Centre, 11 Third Hospital Avenue, Singapore, 168751 Singapore; 5 Duke-NUS Graduate Medical School, Singapore, Singapore

**Keywords:** Cosmesis, Cosmetic outcome, Femtosecond laser, Pterygium, Pterygium surgery

## Abstract

**Background:**

To examine the cosmetic outcome of femtosecond laser-assisted pterygium surgery (FLAPS) with conjunctival autograft (CAG) and its potential predictive factors.

**Methods:**

This was a prospective interventional case series (NCT02866968). We included 29 patients (29 eyes) with primary pterygium who underwent FLAPS. Cosmetic outcome was graded by two graders (an ophthalmology resident and an experienced ophthalmologist) using Hirst classification system (1–4 = excellent–poor). Weighted Cohen’s kappa analysis was performed to examine the intra- and inter-rater reliability. The relationship between cosmetic outcome and various factors were determined by Spearman’s correlation coefficients (*r*).

**Results:**

The preoperative severity of pterygium (Tan grading system) was mild/atrophic (7%), moderate/intermediate (62%), and severe/fleshy (31%). An ultrathin CAG (mean thickness of 74.5 ± 9.8 μm) was fashioned intraoperatively. An excellent cosmetic outcome of FLAPS (median ± IQR) was observed at 3 months (1.0 ± 1.0) and remained similar at 6 months (1.0 ± 0.0) and 12 months (1.0 ± 0.0) postoperatively. At final follow-up, 27 (93%) patients achieved good-to-excellent cosmetic outcome, with 1 (3%) patient having a poor outcome due to incomplete pterygium removal. Weighted kappa analysis of Hirst grading system showed excellent intra-rater (κ = 0.86–0.95) and inter-rater reliability (κ = 0.84–0.88). There was a weak and borderline significant correlation between good cosmetic outcome and reduced postoperative CAG thickness (*r* = 0.38, *P* = 0.06) but not with age, gender, preoperative pterygium severity, or intraoperative CAG thickness.

**Conclusions:**

FLAPS can result in an excellent cosmetic outcome, which may be attributed to the beneficial effect of an ultrathin CAG.

**Trial registration:**

ClinicalTrials.gov, NCT02866968. Registered in July 2016,

**Supplementary Information:**

The online version contains supplementary material available at 10.1186/s40662-021-00230-w.

## Background

Pterygium is a common degenerative ocular surface disease, characterized by an abnormal wing-shaped subconjunctival fibrovascular growth onto the cornea, usually in a centripetal fashion. It is estimated to affect 12% of the global population [1]. The pathogenesis of pterygium is multifactorial, with chronic exposure to ultraviolet-B light being the main risk factor [2]. In addition, localized limbal stem cell failure and inflammation have been implicated in the pathogenesis of pterygium [3, 4]. Depending on the disease severity, it can result in a range of symptoms and signs, including ocular surface irritation, decreased vision, irregular astigmatism, and unappealing cosmesis [5].

Surgery is the mainstay of treatment for symptomatic or cosmetically unacceptable pterygium. Previously, direct removal of pterygium with a bare sclera technique was widely performed due to the ease and speed of the procedure. However, in view of the high recurrence rate (around 38–88%), this technique has been superseded by (extended) removal with conjunctival autograft (CAG), which is now considered the gold standard for managing pterygium in clinical practice [5, 6]. The advantage of a CAG is supported by a recent Cochrane meta-analysis of 1947 eyes, which demonstrated that the recurrence rate of pterygium with CAG was significantly better than with amniotic membrane transplant (3–17% vs. 3–42%) [7].

With the advancement of surgical techniques and reducing recurrence rate, postoperative cosmesis is now emerging as an important patient outcome following pterygium surgery [8–13]. In 2011, Hirst proposed and validated a new web-based pterygium grading system for cosmesis, based on the postoperative conjunctival appearance following Pterygium Extended Removal Followed by Extended Conjunctival Transplantation (*P.E.R.F.E.C.T.* for PTERYGIUM), to help standardize the reporting of cosmetic outcomes [13]. In addition, a number of other classification systems, based on the morphologic appearance of pterygium body [14] and caruncle [15], have been proposed to grade the preoperative severity of pterygium and to predict the “success” of pterygium surgery, which is mainly defined by the rate of recurrence and visual outcome. However, the use of these pterygium grading systems for predicting postoperative cosmetic outcome has not been investigated.

In our recent studies, we demonstrated that femtosecond laser could be employed to fashion a consistently ultrathin CAG during pterygium surgery and achieve a low rate of postoperative complication or recurrence [16, 17]. The reperfusion of CAGs was not affected even when the grafts were ultrathin [18]. However, whether a thinner CAG translates to a better cosmetic outcome remains unknown. In this study, we aimed to examine the postoperative cosmetic outcome of FLAPS and its correlation with various preoperative, intraoperative, and postoperative factors.

## Methods

This study was conducted as a prospective interventional case series (NCT02866968) to evaluate the feasibility and safety of FLAPS. The study was conducted at the Singapore National Eye Centre between July 2016 and June 2017. Informed consent was obtained from all patients prior to surgery and the study protocol was approved by the SingHealth Centralized Institutional Review Board (R1361/47/2016).

### Preoperative assessment using the Tan grading system

The Tan grading system for pterygium was devised based on the preoperative clinical appearance of the pterygium at the slit-lamp, with a given score of T1 (atrophic/mild), T2 (intermediate/moderate), and 3 (fleshy/severe) [14]. Two graders, including a medical intern (A.J.) and a fully qualified ophthalmologist (Y.C.L.), were selected to grade the pterygium using the grading system. The grading was performed independently by each rater and was repeated once more at least 1 month after the first assessment so as to determine the intra- and inter-rater reliability.

### Surgical technique

The surgical technique of FLAPS employed in this study was detailed in our previous publication [19]. All surgeries were performed by a single surgeon (J.S.M.). Briefly, the pterygium head was manually removed from the corneal surface with a Mini-Blade (Beaver-Visitec, Waltham, MA) and the remaining pterygium body and extensive subconjunctival Tenon tissues were removed to expose the bare sclera and reduce the recurrence rate [17]. After measuring the size of conjunctival defect, an ellipsoid, 7 mm × 10 mm CAG of 60 μm depth was fashioned from the superior conjunctiva using a femtosecond laser platform, Z8 (Ziemer Ophthalmic Systems AG, Port, Switzerland), with lamellar keratoplasty module. The mean width and length of the conjunctival defects were 5.8 ± 1.2 mm (and 9.1 ± 1.4 mm (ranged, 5.5–11.0 mm), respectively. The selected CAG size was the largest size that can be created by the current machine and was sufficient to cover the conjunctival defect of all included cases. As the graft was ellipsoid instead of rectangular (which was usually the case for the conjunctival defect), the graft diameter has to be slightly larger than the conjunctival defect to ensure the entire defect can be covered without any significant tension at the conjunctival graft-host junction. The thickness of the harvested CAG was then immediately measured using the built-in optical coherence tomography (OCT) scanner. Once the CAG was completely dissected from the stromal bed, it was transplanted over the bare sclera area and secured in place with fibrin glue (Artiss; Baxter, Westlake Village, CA). A bandage contact lens was inserted at the conclusion of surgery. Postoperatively, all patients received a 4-week course of levofloxacin four times daily (QID) and a 6-week tapering course of topical dexamethasone, starting from QID. The surgical steps of FLAPS are shown in Supplementary Video 1.


**Additional file 1**

### Postoperative assessment of cosmetic outcome and potential predictive factors

The cosmetic outcome of FLAPS was assessed at 3 months, 6 months, and 12 months postoperatively. Patients were excluded from the study if there was no available data on the cosmetic outcome. The cosmesis was graded by two raters, including an ophthalmology resident (Y.F.L) and an experienced ophthalmologist (Y.C.L.), using the Hirst grading system [13]. A score of 1–5 was assigned to grade the cosmesis: 1 – excellent (no visible signs of surgery apart from corneal opacity); 2 – good (generalized conjunctival vascular changes / injection over the whole ocular surface); 3 – fair (localized conjunctival vascular changes / injection at the pterygium surgical site only); 4 – poor (obvious conjunctival scarring, puckering or arrowhead formation); and 5 – ungradable (quality of image not adequate for grading). Ungradable images were excluded from the analysis. Similarly, the grading was performed independently by each rater and was repeated once more at least 1 month after the first assessment so as to determine the intra- and inter-rater reliability.

The assessment of CAG thickness and conjunctival harvest site (CHS) thickness were performed at 1 day, 1 month, 3 months, 6 months, and 12 months postoperatively using an anterior segment optical coherence tomography (AS-OCT; RTVue, Fremont, CA). Correlation of the postoperative cosmetic outcome with age, gender, baseline pterygium severity (based on the Tan grading system), intraoperative CAG thickness, and postoperative CAG thickness was performed.

### Statistical analysis

Statistical analysis was performed using SPSS version 26.0 (IBM SPSS Statistics for Windows, Armonk, NY, USA). Comparison between groups was conducted using Pearson’s Chi-squared or Fisher’s Exact test where appropriate for categorical variables and unpaired T test or Mann-Whitney U test for continuous variables. Normality of data distribution was assumed if the skewness and kurtosis z-values were between − 1.96 and + 1.96 and the Shapiro-Wilk test *p*-value was greater than 0.05. One-way analysis of variance (ANOVA) was performed to analyse the mean differences of two or more independent groups. All continuous data were presented as mean ± standard deviation (SD), median ± interquartile range (IQR), and/or 95% confidence interval (CI). Missing data on cosmesis, CAG thickness and CHS thickness were compensated by last observation carried forward (LOCF) data.

Intra- and inter-rater reliability was measured by weighted Cohen’s kappa analysis and was categorized as follows: none to slight (κ = 0.01–0.20); fair (κ = 0.21–0.40); moderate (κ = 0.41–0.60); substantial (κ = 0.61–0.80); and almost perfect agreement or excellent (κ = 0.81–1.00) [20, 21]. As four ratings were assigned to each parameter (graded by two raters at two different time points), the median values of the four ratings were calculated and used if the κ values were > 0.60 (i.e., in substantial or almost perfect agreement). If the final median value ended up with a .5 decimal value, a third grader (D.S.J.T.) was selected to grade the specific image. Spearman’s rank-order correlation analysis was performed to examine the relationship between the cosmetic outcome and various parameters and was interpreted as weak (*r* = 0.00–0.40), moderate (*r* = 0.41–0.69), and strong (*r* = 0.70–1.00) [22]. *P*-value of less than 0.05 was considered statistically significant.

## Results

### Patient characteristics

A total of 30 eyes of 30 patients were recruited into the trial and underwent FLAPS. One patient was excluded from this study as it was related to a case of recurrent pterygium, with 29 eyes of 29 patients with primary pterygium being included in the final analysis. The mean age was 62.0 ± 10.1 years with an 80% male preponderance. The main ethnic group was Chinese (24, 83%) followed by Malay (2, 7%), Indian (1, 3%), Burmese (1, 3%), and Caucasian (1, 3%). The baseline severity of pterygium was mild, moderate, and severe in 2 (7%), 18 (62%), and 9 (31%) patients, respectively.

### Reliability of Hirst and Tan grading systems

All 29 patients had slit-lamp images available for grading of the cosmetic outcome. None of the captured images were ungradable. A total of 244 assessment (based on 61 slit-lamp images) were performed by two graders for the cosmetic outcome. Weighted Cohen’s kappa analysis of Hirst grading system showed excellent intra-rater reliability (κ = 0.86 for the ophthalmology trainee and κ = 0.95 for the fully qualified ophthalmologist) and excellent inter-rater reliability (κ = 0.84–0.88; Table 1). In addition, weighted Cohen’s kappa analysis of Tan grading system (based on 116 assessment of 29 slit-lamp images by two graders) demonstrated an excellent intra-rater reliability (κ = 0.88–0.89) and substantial inter-rater reliability (κ = 0.66–0.75; Table 1).

**Table 1:** Weighted Cohen’s kappa analysis of Hirst and Tan grading systems

	Intra-rater reliability		Inter-rater reliability	
Hirst grading system	YFL1, YFL2	YCL1, YCL2	YFL1, YCL1	YFL2, YCL2
	0.86 (SE 0.06, CI 0.75–0.98)	0.95 (SE 0.03, CI 0.89–1.00)	0.84 (SE 0.06, CI 0.72–0.96)	0.88 (SE 0.05, CI 0.79–0.99)
Tan grading system	AJ1, AJ2	YCL1, YCL2	AJ1, YCL1	AJ2, YCL2
	0.88 (SE 0.09, CI 0.71–1.00)	0.89 (SE 0.08, CI 0.73–1.00)	0.66 (SE 0.12, CI 0.42–0.91)	0.75 (SE 0.12, CI 0.52–0.98)

AJ and YFL are junior ophthalmology residents; YCL is an experienced ophthalmologist

*SE* = standard error; *CI* = 95% confidence interval

### Cosmetic outcome of FLAPS

**Fig. 1:**
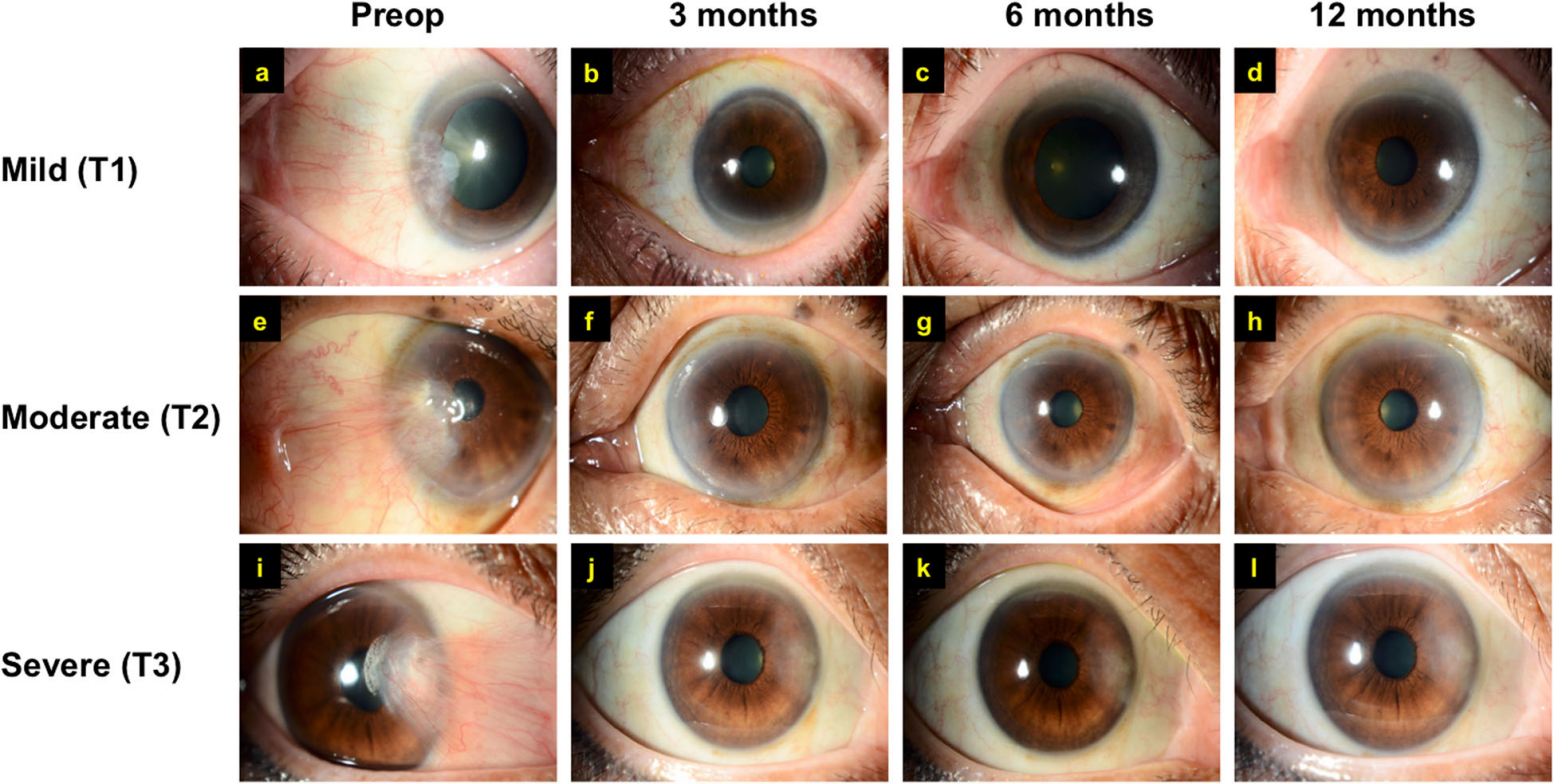
Illustrations of the cosmetic outcomes of patients with different grades of pterygium severity (based on Tan grading system) at various postoperative time points, including early (3 months), intermediate (6 months), and late (12 months) follow-up period. Preoperative and postoperative pictures of the ocular surface of the patient with mild pterygium (**a-d**), moderate pterygium (**e-h**), and severe pterygium (**i-l**). Excellent outcomes were achieved in all these three patients

**Fig. 2:**
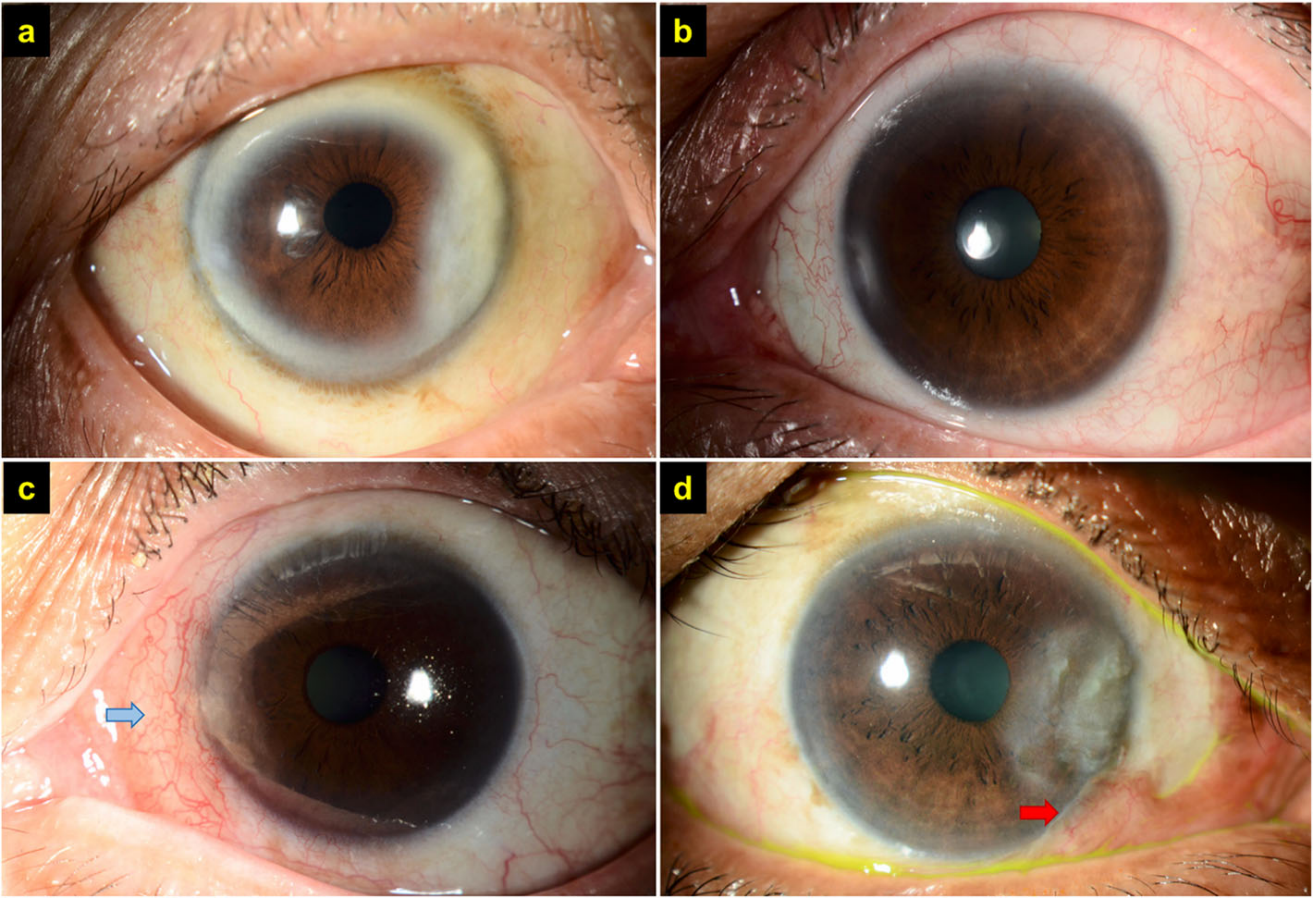
Examples of grading of postoperative cosmetic outcome following femtosecond laser-assisted pterygium surgery. **a** An excellent outcome with a white, normal looking conjunctiva. **b** A good outcome with mild generalized conjunctival vascular injection. **c** A fair outcome with localized conjunctival vascular injection at the surgical site only (*blue arrow*). **d** A poor outcome with localized conjunctival vascular injection and additional conjunctival changes, including conjunctival scarring, puckering and recurrence. In this case, the poor outcome was related to an incomplete removal of the pterygium (*red arrow*)

An excellent cosmetic outcome of FLAPS (median ± IQR) was observed as early as 3 months postoperatively (1.0 ± 1.0), which remained similar at 6 months (1.0 ± 0.0) and 12 months (1.0 ± 0.0) postoperatively (Fig. 1a-l). At the 12-month follow-up, 24 (83%) and 3 (10%) patients achieved excellent and good cosmetic outcome, respectively, with 1 (3%) patient having a poor outcome due to incomplete removal of the inferior pterygium body and poor glue adhesion (Fig. 2a-d). Otherwise, there was no frank recurrence of pterygium noted during the study period. Corneal opacity was observed in some cases post-pterygium removal, though it was not part of the consideration based on the Hirst grading system.

### Predictive factors of cosmetic outcome

Analysis of potential predictive factors of cosmetic outcome, including age, gender, preoperative severity of pterygium, intraoperative CAG thickness, and postoperative CAG thickness, was performed. The mean CAG thickness was 74.5 ± 9.8 μm intraoperatively, 199.9 ± 74.0 μm at 1-day postoperative, and 174.7 ± 86.6 μm at 1-year postoperative, with no significant change observed during the study period (*P* = 0.88; Table 2). Similarly, CHS thickness was 318.9 ± 89.0 μm at 1 day postoperative and reduced to 270.1 ± 77.7 μm at 1 year postoperative, with no significant change over the period (*P* = 0.22; Table 2). There was a weak and borderline significant correlation between good cosmetic outcome and reduced postoperative CAG thickness (*r* = 0.38, *P* = 0.06) but not with age (*P* = 0.12), gender (*P* = 0.18), preoperative pterygium severity (*P* = 0.22), or intraoperative CAG thickness (*P* = 0.62; Table 3).

**Table 2:** Changes in the postoperative conjunctival autograft thickness and conjunctival harvest site thickness following femtosecond laser-assisted pterygium surgery over a 12-month period

	Thickness mean ± SD	% of CAG thickness < 200 μm	*P*-value^a^
Postop CAG thickness (μm)			0.88
1 day (N = 20)	199.9 ± 74.0	50.0	
1 month (N = 23)	187.3 ± 84.6	60.9	
3 months (N = 24)	182.0 ± 87.8	62.5	
6 months (N = 24)	178.5 ± 85.8	58.3	
12 months (N = 24)	174.7 ± 86.6	58.3	
Postop CHS thickness (μm)			0.22
1 day (N = 20)	318.9 ± 89.0	–	
1 month (N = 23)	290.2 ± 82.0	–	
3 months (N = 24)	269.6 ± 88.6	–	
6 months (N = 24)	264.8 ± 84.6	–	
12 months (N = 24)	270.1 ± 77.7	–	

*CAG* = conjunctival autograft; *CHS* = conjunctival harvest site; *SD* = standard deviation

Missing data were compensated by last observation carried forward (LOCF) method

^a^The mean differences of CAG and CHS thickness among different time points were calculated using one-way analysis of variance (ANOVA)

**Table 3:** Correlation of potential predictive factors for good cosmetic outcome following femtosecond laser-assisted pterygium surgery

Dependent variables	Spearman’s correlation coefficients (95% CI)	*P*-value
Age (years)	0.29 (−0.09 to 0.60)	0.12
Gender (female vs. male)	0.25 (−0.13 to 0.58)	0.18
Preop pterygium severity (T1-T3)	0.24 (−0.15 to 0.57)	0.22
Intraop CAG thickness (μm)	−0.10 (−0.45 to 0.29)	0.62
Postop CAG thickness (μm)	0.38 (−0.04 to 0.69)	0.06

*CI* = confidence interval. A positive R value indicates an inverse correlation between the dependent variable and a good outcome and, vice versa, for a negative R value

## Discussion

The success of pterygium surgery has been defined by the rate of recurrence and the visual outcome [14, 23]. With a better understanding of the scientific basis of pterygium and advancement of surgical techniques in recent decades, the clinical outcome of pterygium surgery (particularly with the CAG technique) has significantly improved, with an overall low recurrence rate of 5–20% [5]. This has also resulted in a shift of clinical and research focuses towards cosmetic outcome, which is now considered an important patient reported outcome.

### Cosmetic outcome

In this prospective interventional case series, we demonstrated that FLAPS could yield an excellent cosmetic outcome as early as 3 months postoperative, even in eyes with severe fleshy pterygium. Up to 93% of our patients with primary pterygium achieved a good-to-excellent cosmetic outcome and 3% had a poor outcome due to an incomplete primary removal of pterygium at final follow-up. Otherwise, we did not observe any frank recurrence of pterygium in our study. These findings are superior to the results of pterygium surgery with manually harvested CAG, based on the same grading system [8, 23]. Kucukerdonmez et al. [11] and Prabhasawat et al. [23] previously compared the cosmetic outcome of CAG transplantation and amniotic membrane transplantation (AMT) for pterygium surgery. Based on the Hirst grading system (as described above), a poor cosmetic outcome was reported to be 9–10% and 26–33% in the CAG and AMT groups, respectively [11, 23].

### Reliability of Hirst grading system

A number of grading systems for cosmesis following pterygium surgery have been reported in the literature [8, 13, 23]. However, only the Hirst classification system has undergone a robust validation by 12 graders, including 6 ophthalmologist and 6 non-ophthalmologists, and demonstrated strong intra- and inter-rater reliability and user-friendliness of the system [13]. Therefore, this classification was used for the grading of cosmetic outcome in our study. That said, the reliability of this grading system has not been examined or validated by other research groups. Based on 244 assessment of 61 slit-lamp images (derived from a mixed ethnicity population) graded by a junior ophthalmology resident and an experienced ophthalmologist, we observed an excellent intra- and inter-rater reliability, highlighting the consistency and versatility of this grading system.

### Potential effect of CAG thickness on the cosmetic outcome

To date, the *P.E.R.F.E.C.T* technique proposed by Hirst has one of the highest success rates reported in the field of pterygium surgery, with 0.1% recurrence rate noted in 1000 cases [6]. In addition, an excellent cosmetic outcome can be achieved with this technique, with only 6% cases with poor cosmesis. These favourable clinical and cosmetic outcomes were hypothesized to be related to the extended removal of the subconjunctival Tenon’s tissues [6]. If subconjunctival Tenon’s tissue is not completely removed from the affected site (particularly from the limbal area) or inadvertently incorporated during the harvesting of CAG, and transplanted over the area of bare sclera, the Tenon’s tissues may serve as an undesired source for promoting postoperative inflammation and potential recurrence of pterygium. Hirst noted that 3 (0.3%) of his patients required repeat surgery due to persistent hyperaemia of the CAG, which was likely related to a thick graft with unintended incorporation of Tenon’s tissues. This issue can be reduced or potentially eliminated with the use of femtosecond laser technology. The thickness of conjunctiva, consisting of epithelium and stroma, is age-dependent (i.e., decreases with age) and the average thickness was reported to be around 240.1 ± 29.8 μm (range, 140–304 μm) [24]. In our study, the mean intraoperative CAG thickness was 74.5 ± 9.8 μm, suggesting that only conjunctiva and no subconjunctival Tenon’s tissues were included during the harvesting of CAG, which might account for the excellent cosmetic outcome of FLAPS. In addition, as demonstrated by the relatively consistent intraoperative CAG thickness, FLAPS could potentially reduce the surgeon-surgeon inter-variability in the preparation of CAG, which often serves as an important determinant for postoperative outcome [25].

### Predictors of cosmetic outcome following pterygium surgery

In principle, the Hirst grading system evaluates the cosmetic outcome of pterygium surgery based on the severity of conjunctival hyperaemia or recurrence, which is often the precursor of a frank pterygium recurrence. Therefore, factors that have been shown to increase the risk of recurrence, including younger age, fleshy and higher-grade pterygium, recurrent pterygium, and use of sutures [5, 25–27], could similarly affect the cosmetic outcome of pterygium surgery.

In our study, we did not observe any correlation between the cosmetic outcome of pterygium and various parameters, including age, gender, preoperative severity, and intraoperative CAG thickness. However, the correlation analysis is likely limited by the fact that most (93%) patients achieved a good-to-excellent outcome. One way to study the cause-effect of intraoperative CAG thickness on the clinical and cosmetic outcomes would be to compare the CAG thickness and cosmetic outcomes of two different harvesting methods of CAG [e.g., FLAPS (with ultrathin CAG) versus conventional manual dissection (with standard CAG thickness)] and to follow-up the patients over time. We also observed a weak and borderline significant correlation between a good cosmetic outcome and reduced CAG thickness postoperatively. This is likely due to the fact that the cosmetic outcome was directly related to the degree of postoperative conjunctival inflammation, which could influence the swelling or thickness of the CAG. Other factors such as the extent and thoroughness of tenonectomy and the use of fibrin glue (instead of sutures) may also have a positive influence on the cosmetic outcome. Interestingly, the severity of pterygium (based on the Tan grading system) did not have any significant influence on the cosmetic outcome. However, the Tan system was previously devised and shown to predict the risk of recurrence in bare scleral surgery, which is no longer a standard practice in most regions. With this in mind, we have recently proposed and validated a new pterygium grading system, named SLIT2, with an aim to improve and standardize the grading of pterygium in the current practice [28].

In this FLAPS technique, we advocate a sutureless approach with fibrin glue in view of the lower risk of recurrence, speed and ease of procedure, and better patient comfort. In addition, it would be more technically challenging to suture an ultrathin graft compared with gluing. Based on a recent meta-analysis [26], a poor cosmetic outcome (using recurrence as an indicator) was observed in at least 4.3 and 9.9% in the fibrin glue and sutured groups, respectively. Moreover, we did not observe any CAG dislocation in any of our patients during the study period, suggesting that fibrin glue is a safe technique for securing ultrathin CAGs.

### Strengths and limitations

We acknowledge that this prospective interventional case series was limited by the small sample size and constrained to a single-surgeon experience. However, the favourable cosmetic outcome observed in this study lends strength to further research exploration and potential clinical application. It is also noteworthy to mention that most of our patients achieved good-to-excellent cosmetic outcome, which limited the correlation analysis. Ocular cosmesis is known to have significant psychosocial effect on the affected person [29, 30], and therefore the long-term positive cosmetic and clinical outcome of FLAPS, shown in this study, could have considerable impact on the patients. In addition, we did not observe any risk of recurrence in our study and our ongoing audit of the FLAPS (> 200 cases now) showed that the risk of recurrence is less than 1% (unpublished observation). Further studies comparing FLAPS and other techniques such as standard pterygium surgery with CAG and *P.E.R.F.E.C.T* will be clinically beneficial. Another aspect that was not evaluated in this study was the effect of postoperative corneal opacity on the cosmetic outcome as it was not part of the Hirst grading system. It would be valuable to explore whether this is an important aspect of the cosmetic outcome from the patient’s perspective, which helps to determine the need for its integration with the current cosmetic grading system after pterygium surgery.

As FLAPS is dependent on the femtosecond laser technology, which is associated with a high cost, comparison of the outcome and cost-effectiveness of FLAPS with other surgical approaches would be valuable in the future. However, when considering the cost and cost-effectiveness, it is important to take into account the versatility of the femtosecond laser technology as the same femtosecond laser technology can also be employed for other clinical applications such as creation of LASIK flaps, cataract surgery and corneal transplantation, which allows the cost-effectiveness of this technology to be maximized [31–33].

In conclusion, an excellent cosmetic outcome can be achieved at short- and long-term time points following FLAPS. An ultrathin CAG can be consistently fashioned by femtosecond laser and may have a beneficial effect on cosmetic outcomes.

## Data Availability

All data have been provided in this study.
